# Pathologic complete response and overall survival in breast cancer subtypes in stage III inflammatory breast cancer

**DOI:** 10.1007/s10549-019-05219-7

**Published:** 2019-04-10

**Authors:** Dominique J. P. van Uden, Marissa C. van Maaren, Peter Bult, Luc J. A. Strobbe, J. J. M. van der Hoeven, Charlotte F. J. M. Blanken-Peeters, Sabine Siesling, Johannes H. W. de Wilt

**Affiliations:** 1 Department of Surgery, Canisius Wilhelmina Hospital, Weg door Jonkerbos 100, 6532 SZ Nijmegen, The Netherlands; 2 Department of Research, Netherlands Comprehensive Cancer Organisation (IKNL), Hoog Catharijne, Godebaldkwartier 419, 3511 DT Utrecht, The Netherlands; 3 Department of Health Technology and Services Research, University of Twente, Drienerlolaan 5, 7522 NB Enschede, The Netherlands; 4 Department of Pathology, Radboud University Medical Center Nijmegen, Geert Grooteplein Zuid 10, 6525 GA Nijmegen, The Netherlands; 5 Department of Medical Oncology, Radboud University Medical Center Nijmegen, Geert Grooteplein Zuid 10, 6525 GA Nijmegen, The Netherlands; 6 Department of Surgery, Rijnstate Hospital, Wagnerlaan 55, 6815 AD Arnhem, The Netherlands; 7 Department of Surgical Oncology, Radboud University Medical Center Nijmegen, Geert Grooteplein Zuid 10, 6525 GA Nijmegen, The Netherlands

**Keywords:** Inflammatory breast cancer, Breast cancer subtypes, Survival, Epidemiology

## Abstract

**Purpose:**

To analyze the influence of hormone receptors (HR) and Human Epidermal growth factor Receptor-2 (HER2)-based molecular subtypes in stage III inflammatory breast cancer (IBC) on tumor characteristics, treatment, pathologic response to neoadjuvant chemotherapy (NACT), and overall survival (OS).

**Methods:**

Patients with stage III IBC, diagnosed in the Netherlands between 2006 and 2015, were classified into four breast cancer subtypes: HR+/HER2− , HR+/HER2+ , HR−/HER2+ , and HR−/HER2− . Patient-, tumor- and treatment-related characteristics were compared. In case of NACT, pathologic complete response (pCR) was compared between subgroups. OS of the subtypes was compared using Kaplan–Meier curves and the log-rank test.

**Results:**

1061 patients with stage III IBC were grouped into subtypes: HR+/HER2− (*N *= 453, 42.7%), HR−/HER2− (*N *= 258, 24.3%), HR−/HER2+ (*N *= 180,17.0%), and HR+/HER2+ (*N *= 170,16.0%). In total, 679 patients (85.0%) received NACT. In HR−/HER2+ tumors, pCR rate was highest (43%, (*p* < 0.001). In case of pCR, an improved survival was observed for all subtypes, especially for HR+/HER2+ and HR−/HER2+ tumor subtypes. Trimodality therapy (NACT, surgery, radiotherapy) improved 5-year OS as opposed to patients not receiving this regimen: HR+/HER2− (74.9 vs. 46.1%), HR+/HER2+ (80.4 vs. 52.6%), HR−/HER2+ (76.4 vs. 29.7%), HR−/HER2− (47.6 vs. 27.8%).

**Conclusions:**

In stage III IBC, breast cancer subtypes based on the HR and HER2 receptor are important prognostic factors of response to NACT and OS. Patients with HR−/HER2− IBC were less likely to achieve pCR and had the worst OS, irrespective of receiving most optimal treatment regimen to date (trimodality therapy).

## Introduction

Inflammatory breast cancer (IBC) is the most aggressive form of breast cancer, and is associated with a dismal prognosis compared to non-IBC in patients with and without distant metastases [1, 2]. At presentation, almost 80% of IBC patients have lymph node involvement and nearly 40% present with distant metastases [3], of which lungs, liver, brain, and skeleton are the most frequent sites [4].

In breast cancer, four subtypes have been identified based on the hormone receptors (HR) [estrogen receptor (ER), progesterone receptor (PR)] and Human Epidermal growth factor Receptor-2 (HER2) [5]. These subtypes include HR+/HER2− (luminal A), HR+/HER2+ (luminal B), HR−/HER2− (triple negative), and HR−/HER2+ (HER2-enriched) [6].

In non-IBC, HER2-enriched and triple-negative tumors have increased breast cancer-specific mortality in comparison with the other subtypes [7, 8]. For IBC, a recent analysis of the American National Cancer Database showed that HR-positive disease was not associated with an advantageous prognosis, and HER2-enriched status was not correlated with unfavorable overall survival (OS). Triple-negative and HR+/HER2− subtypes both showed significantly worse survival compared to the other subgroups [9].

Current treatment of stage III IBC includes neoadjuvant chemotherapy (NACT), surgery, and adjuvant locoregional radiotherapy (trimodality therapy). Moreover, (neo)adjuvant trastuzumab and (neo)adjuvant endocrine therapy are applied in patients with HER2-positive and/or HR-positive tumors, respectively. This has positively influenced the survival of IBC patients in recent years [3]. Sensitivity to NACT might be used as an indicator for survival, since pathologic complete response (pCR) is a predictor of favorable long-term outcome [10]. In non-IBC, the incidence and prognostic impact of pCR varies among breast cancer subtypes [11]. HER2-enriched and triple-negative tumors are more likely to achieve pCR after neoadjuvant systemic therapies, which improves survival in these subgroups [12]. However, patients with residual disease have significantly worse survival if they have triple-negative breast cancer [11].

Due to the low incidence, and variety in case definition, data about IBC are scarce and diverse [13]. Furthermore, several population-based studies regarding IBC have presented contradictory results concerning the association of HR status and prognosis [9, 14]. These inconsistent results might be due to limited sample sizes in single-center or small multicenter studies [15], comparison of populations treated in different eras before the availability of anti-HER2-targeted therapy [14], or the lack of information on systemic therapy in general [9]. We examined the significance of HR/HER2-based subtypes in stage III IBC on clinicopathological characteristics, pCR, and OS in a nationwide patient cohort, selected from the Netherlands Cancer Registry (NCR), in which the TNM-based definition of IBC has not changed over time with available information on systemic and targeted therapy.

## Materials and methods

### Data source

The NCR is a nationwide population-based cancer registry. All newly diagnosed malignancies in the Netherlands are recorded and notified through the nationwide Pathology Archive (PALGA), which collects all pathology reports of Dutch hospitals (*N *= 92). Trained NCR registrars directly collect patient-, tumor- and treatment-related characteristics from the patient’s medical records. The NCR collects cancer incidence data covering more than 95% of all cancer patients in the Netherlands [16]. Morphology and differentiation are graded according to the International Classification of Diseases for Oncology [17]. Staging is performed according to the TNM classification [18, 19]. With respect to IBC, the TNM criteria have not changed over time. The municipal administration was used to verify the patient’s vital status and, if applicable, date of death through a yearly linkage. Follow-up has been completed until February 29, 2016. The privacy committee of the NCR has approved this study.

### Patients and study variables

Patients, diagnosed from 2006 to 2015, with clinical T4dN0–3M0 breast cancer were identified, excluding patients with only a pathological T4d status without clinical T4d status.

The year 2006 was selected as the start of the analysis since HER2 was not routinely collected prior to 2006. Histological type and HR/HER2 status were assessed in the primary tumor from a needle biopsy prior to any therapy. Patients were classified into four breast cancer subtypes, based on HR status and HER2 status: HR+ (ER+ and/or PR+)/HER2− , HR+ (ER+ and/or PR+)/HER2+, HR− (ER− and PR−)/HER2+ (HER2-enriched), and HR− (ER− and PR−)/HER2− (triple negative). Patients were excluded when data on HR and/or HER2 receptor status were missing.

According to Dutch guidelines, ER/PR status had been determined with immunohistochemistry (IHC). At least 10% positive tumor nuclei were considered as a positive result. In the Netherlands, HER2 status was considered positive with an immunohistochemical score of 3 + (at least 10% of tumor cells with strong complete membrane staining) or amplification of the HER2 gene diagnosed with in situ hybridization (ISH) (in at least 10% of tumor cells showing a ratio of HER2 probe to centromere chromosome 17 probe of > 2.2 or with single probe HER2 test when mean > 6 HER2 genes per tumor nucleus were detected) or with other amplification-based techniques, such as multiplex ligation-dependent probe amplification (MLPA). In case of an immunohistochemical score of 2 + (at least 10% of tumor cells with slight to moderate complete membrane staining, considered as an equivocal result), ISH or MLPA was performed. If in this case HER2 was found to be amplified, HER2 was considered positive. HER2 status was considered negative with an immunohistochemical score of 0 or 1 + or if ISH or MLPA showed no amplification of the HER2 gene. In the Netherlands, some variation in determining the HER2 status existed in the period 2006–2016 (especially the cutoff for amplification (> 2,2 or ≥ 2) in the double probe ISH test). For this study the HER2 status as was registered in the NCR was used.

Compared to other authors, we did not incorporate tumor grade in the subtype classification [9].

If data were missing for pretreatment biopsies, this was substituted with data of the postoperative specimen. In all cases, the highest known grade was registered. Grade could be determined in 99% of patients who underwent a surgical procedure without NACT. If available, clinical lymph node involvement was determined on clinical and radiological findings and pretreatment pathological information. Pathological lymph node involvement was determined after surgery without NACT (pN status) or with NACT (ypN status). Trimodality treatment (NACT, surgery, and adjuvant locoregional radiation therapy) was analyzed. Chemotherapy, endocrine therapy, and targeted therapy (trastuzumab) were reported as administered or not administered. If available, specific types of chemotherapy were assessed. Pathological response to NACT was analyzed and pCR was defined as the absence of microscopic residual invasive cancer in the surgically removed specimen (ypT0 and ypTis) after NACT, whereas the absence of invasive residual disease in axillary lymph nodes was considered as lymph node pCR (ypN0). With respect to tumor stage, ypT1-3 tumors were considered to have achieved a partial response to NACT. Resection margin status was reported (from 2011 onwards) as free, focal incomplete, or more than focal incomplete, according to the Dutch Guideline for Breast Cancer Treatment. A free resection margin is defined as no tumor, either invasive and/or ductal carcinoma in situ (DCIS), reaching the inked margin. No information was available concerning re-operations.

### Statistical analysis

Tumor characteristics were compared between the different subtypes using Chi-squared tests for categorical variables and non-parametric approaches (Mann–Whitney *U* tests) for continuous variables. Fisher’s exact test was used to determine if there were non-random associations between two categorical variables in case of less than five patients per stratum. In case of too little events to calculate the *p* value accurately, the *p* value was not calculated. The OS was calculated from the date of diagnosis to the date of death from any cause, or the last date of observation. Follow-up was calculated until time of death or end of observation. OS was determined using Kaplan–Meier curves and breast cancer subtypes were compared using the log-rank test. To adjust for patient-, tumor-, and treatment-related characteristics, a multivariable Cox proportional hazard analysis was performed. Variables included were age, breast cancer subtype, nodal stage, histological tumor type, and grade, and trimodality therapy, as these variables were significantly different between breast cancer subtypes and significantly influenced the outcome (*p* < 0.1). For all other analyses, a *p* value < 0.05 was considered as statistically significant. All statistical analyses were performed in the software package STATA version 14.1.

## Results

### Patient characteristics

From 2006 to 2015, 1216 patients with stage III IBC were identified. After exclusion of 114 patients due to unknown HR/HER2 status and 41 patients with a pathological T4d status without clinical T4d status, 1061 patients remained for analysis. Clinicopathological characteristics for each subtype are shown in Table 1.

**Table 1:** Clinicopathological characteristics of all stage III IBC patients from 2006–2015, by breast cancer subtype

	HR+/HER2− (*N *= 453)	HR+/HER2+ (*N *= 170)	HR−/HER2+ (*N *= 180)	HR−/HER2− (*N *= 258)	*p* value
Clinicopathological characteristics					
Mean age (SD)	63.2 (16.1)	58.4 (16.2)	60.0 (16.4)	60.7 (15.3)	0.004
Histological subtype					
Ductal	362 (80.0)	152 (89.4)	168 (93.3)	225 (87.2)	< 0.001
Lobular	64 (14.1)	8 (4.7)	4 (2.2)	14 (5.4)	
Other	27 (6.0)	10 (5.9)	8 (4.4)	19 (7.4)	
Grade					
1–2	109 (24.0)	20 (11.8)	12 (6.7)	18 (7.0)	< 0.001
3	59 (13.0)	26 (15.3)	46 (25.6)	97 (37.6)	
Unknown	285 (63.0)	124 (72.9)	122 (67.8)	143 (55.4)	
cN-stage					
Node negative	61 (13.5)	43 (25.3)	60 (33.3)	60 (23.3)	< 0.001
Node positive	308 (68.0)	100 (58.8)	104 (57.8)	174 (67.4)	
Unknown	84 (18.5)	27 (15.9)	16 (8.9)	24 (9.3)	
Resection margin status^$^					
Free	169 (90.8)	89 (97.8)	95 (97.9)	108 (92.3)	0.071
Focally incomplete	10 (5.4)	0 (0.0)	1 (1.0)	3 (2.6)	
More than focal incomplete	7 (3.8)	2 (2.2)	1 (1.0)	6 (5.1)	

*cN*-*stage* clinical lymph node stage, *NFS* not further specified, *HR* hormone receptor, *HER2* Human Epidermal growth factor Receptor-2, *SD* standard deviation

^$^At time of pathological analysis after surgical removal of the tumor

### Histopathological and molecular characteristics

The distribution of breast cancer subtypes was as follows: HR+/HER2− (*N *= 453, 42.7%), HR−/HER2− (triple negative) (*N *= 258, 24.3%), HR−/HER2+ (HER2-enriched) (*N *= 180, 17.0%), HR+/HER2+ (*N *= 170, 16.0%). In the HR+/HER2− and HR+/HER2+ groups, 14 and 12 tumors, respectively, were ER− and PR+ .

Invasive ductal carcinoma was the most common type of breast cancer (85.5%) regardless of breast cancer subtype. Compared to the other groups, patients with HR−/HER2+ tumors were more likely to have a higher grade (*p* < 0.001). In 35% of patients operated following NACT, grade was determined pre-NACT.

### Treatment of IBC

In 670 patients (63.1%), NACT was administered and least often in HR+/HER2− IBC. Adjuvant radiotherapy was given in 679 patients (66.4–78.3%; 85.0% of all operated patients). Adjuvant radiotherapy was administered less often in HR+/HER2− IBC (60.9%), compared to HER2-enriched (66.7%), HR−/HER2− (66.3%), and HR+/HER2+ (65.9%) IBC (Table 2).

**Table 2:** Treatment characteristics of all stage III IBC patients from 2006–2015, by breast cancer subtype

Treatment characteristics					
Surgical procedure	318 (70.2)	135 (79.4)	146 (81.1)	200 (77.5)	
Type of surgery					
BCT − ALND	6 (1.3)	4 (2.4)	1 (0.6)	1 (0.4)	0.086
BCT + ALND	7 (1.6)	3 (1.8)	2 (1.1)	4 (1.6)	
MAST − ALND	46 (10.2)	26 (15.3)	8 (4.4)	23 (8.9)	
MAST + ALND	257 (56.7)	101 (59.4)	134 (74.4)	170 (65.9)	
Surgery, NFS	1 (0.2)	0 (0.0)	1 (0.6)	2 (0.8)	
ALND only	1 (0.2)	1 (0.6)	0 (0.0)	0 (0.0)	
Radiotherapy					
No	152 (33.6)	49 (28.8)	48 (26.7)	56 (21.7)	0.006
Neoadjuvant	3 (0.7)	0 (0.0)	0 (0.0)	4 (1.6)	
Adjuvant	276 (60.9)	112 (65.9)	120 (66.7)	171 (66.3)	
Radiotherapy, no surgery	22 (4.9)	9 (5.3)	12 (6.7)	27 (10.4)	
Chemotherapy					
No	166 (36.6)	42 (24.7)	24 (13.3)	45 (17.4)	<0.001
Neoadjuvant	243 (53.6)	113 (66.5)	124 (68.9)	165 (64.0)	
Neoadjuvant and adjuvant	7 (1.6)	3 (1.8)	4 (2.2)	11 (4.3)	
Adjuvant	14 (3.1)	3 (1.8)	5 (2.8)	6 (2.3)	
Chemotherapy, no surgery	23 (5.1)	9 (5.3)	23 (12.8)	31 (12.2)	
Type of chemotherapy					
Anthracyclines	16 (5.6)	2 (1.5)	6 (3.8)	14 (6.7)	<0.001
Taxanes	67 (23.4)	55 (43.0)	60 (38.5)	58 (27.2)	
Anthracyclines + taxanes	131 (45.6)	43 (33.6)	29 (18.6)	90 (42.2)	
NFS	67 (25.4)	28 (21.9)	61 (39.1)	51 (23.9)	
Endocrine therapy					
No	42 (9.3)	22 (12.9)	173 (96.1)	247 (95.7)	<0.001
Neoadjuvant	49 (10.8)	21 (12.3)	1 (0.6)	1 (0.4)	
Neoadjuvant and adjuvant	10 (2.2)	6 (3.5)	0 (0.0)	0 (0.0)	
Adjuvant	229 (50.5)	90 (52.9)	4 (2.2)	5 (1.9)	
Endocrine therapy, no surgery	123 (27.2)	31 (18.2)	2 (1.1)	5 (1.9)	
Targeted therapy					
No	448 (98.9)	41 (24.1)	31 (17.2)	255 (98.8)	<0.001
Neoadjuvant	2 (0.4)	64 (37.6)	76 (42.2)	3 (1.2)	
Neoadjuvant and adjuvant	0 (0.0)	26 (15.3)	29 (16.1)	0 (0.0)	
Adjuvant	1 (0.2)	29 (17.1)	24 (13.3)	0 (0.0)	
Targeted therapy, no surgery	2 (0.4)	10 (5.9)	20 (11.1)	0 (0.0)	
Trimodality therapy					
No	234 (51.7)	78 (45.9)	74 (41.1)	115 (44.6)	0.068
Yes	219 (48.3)	92 (54.1)	106 (58.9)	143 (55.4)	

*BCT* breast-conserving therapy, *MAST* mastectomy, *ALND* axillary lymph node dissection, *NFS* not further specified, *HR* hormone receptor, *HER2* Human Epidermal growth factor Receptor-2

Of the 623 patients with HR-positive (HR+/HER2– and HR+/HER2+) IBC, 559 patients (89.7%) received antihormonal treatment. In patients with HR+/HER2+ and HR−/HER2+ disease (350 patients), trastuzumab was perioperatively administered in 248 patients (70.9%).

Surgery was performed in 799 patients (75.3%), of which mastectomy with axillary lymph node dissection was performed most often in all subgroups (56.7–74.4%). Twenty-eight patients underwent breast-conserving therapy and in 24 of these patients information concerning surgical margins was available: 21 patients had a complete and 3 patients underwent an incomplete resection.

In 560 (52.8%) of all IBC patients (and in 78.0% of patients who were surgically treated), trimodality therapy was applied, regardless of subgroups.

Patients receiving NACT were younger (54.0 years ± 11.8 years) compared to patients not receiving it (73.7 years ± 14.6 years). The median age for HER2+ patients receiving trastuzumab was 54.6 years ± 13.7 years, compared with 63.7 years ± 16.1 years for patients not receiving it.

### Response to neoadjuvant chemotherapy

pCR of the breast tumor was achieved in 156 patients (23.2%), out of 670 patients who received NACT. pCR of the breast and lymph nodes was reported in 10 of 250 patients with HR+/HER2− (4.0%), 24 of 116 patients with HR+/HER2+ (20.7%), 47 of 128 patients with HR−/HER2+ (36.7%), and 28 of 176 patients with HR−/HER2− (15.9%) (Table 3).

**Table 3:** Pathologic response to neoadjuvant chemotherapy for the primary breast tumor and axillary lymph node status in patients with stage III IBC receiving neoadjuvant chemotherapy

	cT4d	ypT0	ypTis	ypT1-3	ypT4	Unknown
Tumor stage response						
HR+/HER2−	250	24 (9.6)	2 (0.8)	159 (63.6)	37 (14.8)	28 (11.2)
HR+/HER2+	116	33 (28.5)	2 (1.7)	62 (53.4)	7 (6.0)	12 (10.3)
HR−/HER2+	128	55 (43.0)	14 (10.9)	41 (32.0)	8 (6.3)	10 (7.8)
HR−/HER2−	176	44 (25.0)	4 (2.3)	90 (51.1)	25 (14.2)	13 (7.4)
Total	670	156 (23.2)	22 (3.2)	352 (52.5)	77 (11.5)	63 (9.4)
Nodal stage response	cN+	ypN0	ypN1-3	Unknown		
HR+/HER2−	192 (35.0)	23 (12.0)	165 (85.9)	4 (2.1)		
HR+/HER2+	96 (17.5)	40 (41.7)	45 (46.9)	11 (11.5)		
HR−/HER2+	108 (19.7)	60 (55.6)	46 (42.6)	2 (1.9)		
HR−/HER2−	152 (27.7)	43 (28.3)	102 (67.1)	7 (4.6)		
Total	548	166 (30.3)	358 (65.3)	24 (4.4)		
Combined response	ypT0/TisN0					
HR+/HER2−	10 (4.0)					
HR+/HER2+	24 (20.7)					
HR−/HER2+	47 (36.7)					
HR−/HER2−	28 (15.9)					
Total	109 (100.0)					

In patients receiving trimodality therapy, achieved pCR rates (after NACT and surgery and prior to adjuvant radiation therapy) were HR+/HER2− 14 (6.4%), HR+/HER2+ 22 (23.9%), HR−/HER2+ 47 (44.3%), HR−/HER2− 28 (19.6%).

### Survival outcomes

**Fig. 1:**
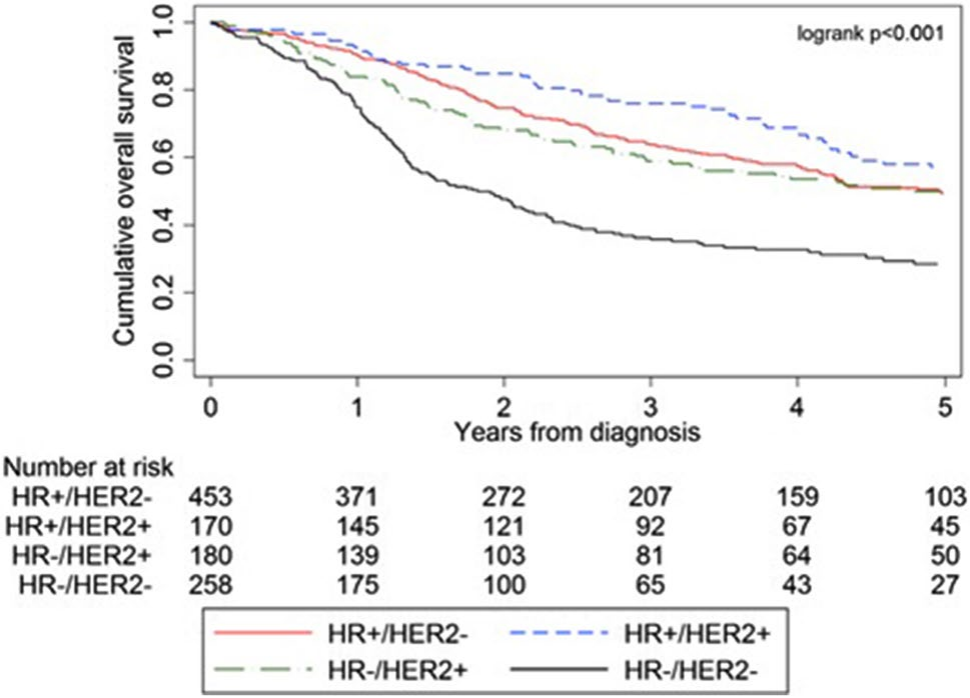
Survival curves for stage III IBC per breast cancer subtype, diagnosed from 2006–2015. Abbreviations: HR, hormone receptor; HER2, Human Epidermal growth factor Receptor-2

Figure 1 shows crude OS of all included patients, subdivided by subtype. The study cohort had a median survival of 4.2 years with a median follow-up of 2.4 years (interquartile range 1.1–4.5) and a 5-year OS rate of 55.6%. OS differed significantly between the subtypes (*p* < 0.001), with the worst OS for triple-negative IBC: HR+/HER2− (60.0%), HR+/HER2+ (67.7%), HR−/HER2+ (57.2%), HR−/HER2− (38.8%). After correction for confounding variables, HR−/HER2− disease still was associated with a significantly lower 5-year OS, compared to HR+/HER2− disease [HR 2.40 (95% CI 1.29–4.45)] (Table 4).

**Table 4:** Adjusted hazard ratios for 5-year overall survival in IBC patients according to breast cancer subtype and OS rates of HR/HER2 defined subtypes in patients with or without pCR following neoadjuvant therapy

Subtype	Hazard ratio (95% CI)^a^	*p* value
Adjusted hazard ratios for 5-year OS		
HR+/HER2−	1 (reference)	
HR+/HER2+	1.55 (0.71–3.40)	0.266
HR−/HER2+	1.70 (0.75–3.85)	0.202
HR−/HER2−	2.40 (1.29–4.45)	0.006

	pCR	Non-pCR
5-year OS rates (%) of different HR/HER2-defined subtypes in case of pCR of non-pCR		
HR+/HER2−	80.0	70.6
HR+/HER2+	91.7	72.4
HR−/HER2+	83.0	50.0
HR−/HER2−	57.1	42.5

^a^Variables included in the final multivariable model were age, breast cancer subtype, nodal stage, histological tumor type, tumor grade, trimodality therapy, pathologic complete response, and partial response to neoadjuvant systemic therapy. Abbreviations: OS, overall survival; HR, hormone receptor; CI, confidence interval; HER2, human epidermal growth factor receptor 2

Overall, patients who received NACT had better OS compared to those who did not: 68.2% versus 34.0% (*p* < 0.001).

**Fig. 2:**
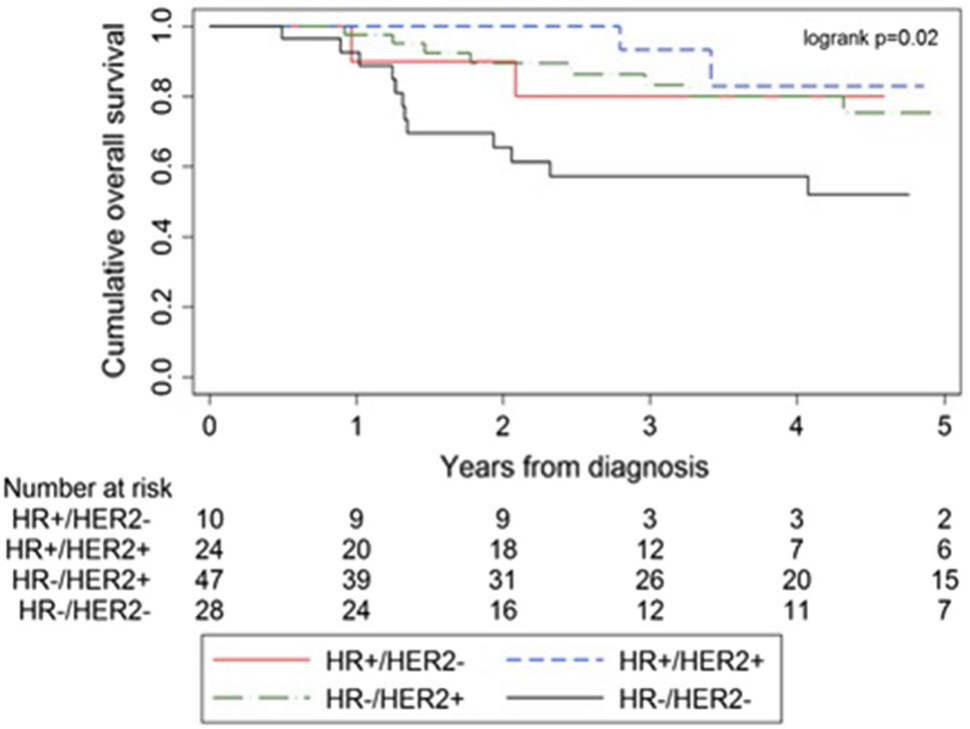
Survival curves for stage III IBC (diagnosed in 2006–2015) per molecular subtype, with clinical positive axillary lymph nodes who reached ypT0/TisN0 after neoadjuvant chemotherapy. Abbreviations: HR, hormone receptor; HER2, Human Epidermal growth factor Receptor-2

Figure 2 displays OS of the different subtypes in case of pCR.

In case of a combined pCR (ypT0/TisN0), an improved survival was observed for all subtypes, but especially for HER2+ tumor subtypes: HR+/HER2− (80.0 vs. 70.6%), HR+/HER2+ (91.7 vs. 72.4%), HR−/HER2+ (83.0 vs. 50.0%), HR−/HER2− (57.1 vs. 42.5%) (Table 4).

A total of 68 patients underwent NACT and (any type of) surgery without subsequent radiotherapy.

Patients receiving subsequent radiotherapy displayed an improved 5-year OS as opposed to patients not receiving radiotherapy: 69.6 versus 55.9% (*p* = 0.021).

**Fig. 3:**
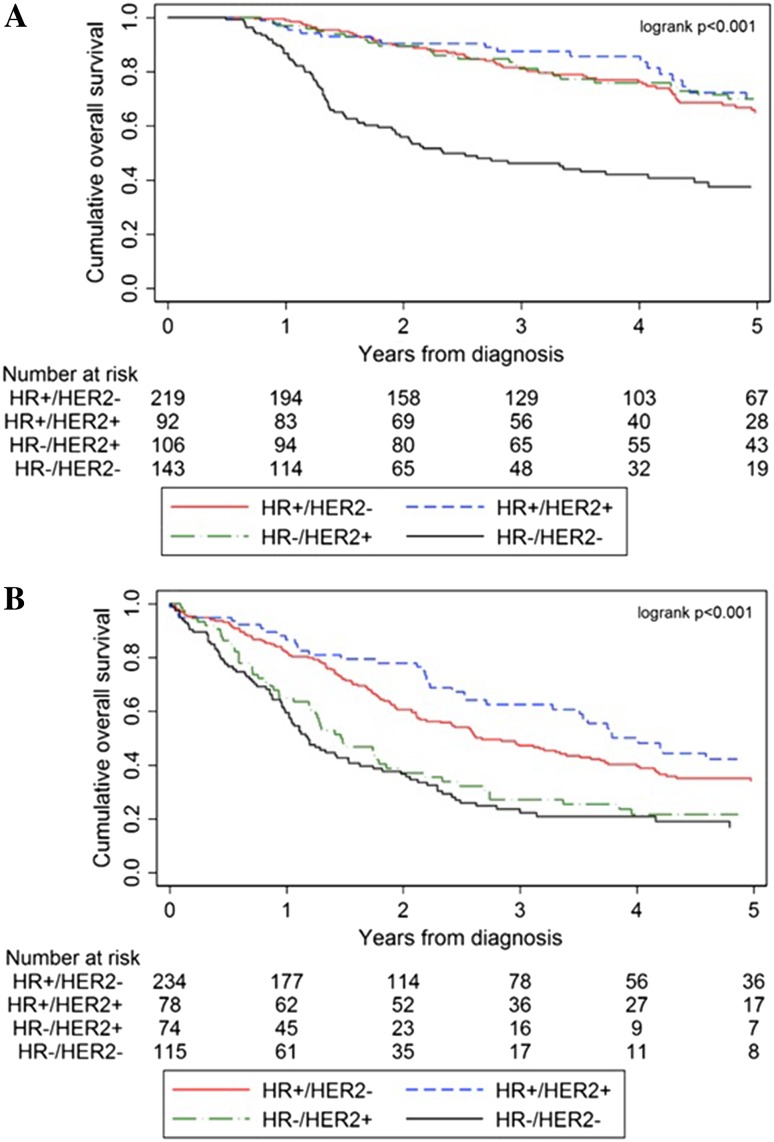
**a**, **b**. Survival curves for stage III IBC (diagnosed in 2006–2015), comparing patients treated with trimodality therapy (**a**), compared to patients with T4d-breast cancer who did not receive trimodality therapy (**a**). Abbreviations: HR, hormone receptor; HER2, Human Epidermal growth factor Receptor-2

Patients who received trimodality therapy had better OS as opposed to those who did not have it (Fig. 3a, b, respectively): HR+/HER2− (74.9 vs. 46.1%), HR+/HER2+ (80.4 vs. 52.6%), HR−/HER2+ (76.4 vs. 29.7%), HR−/HER2− (47.6 vs. 27.8%). After correction for age, breast cancer subtype, morphology, tumor grade, and clinical nodal status, trimodality therapy was still associated with improved survival [HR 0.42 (95% CI 0.29–0.59)].

## Discussion

Our study, the largest to date evaluating the impact of breast cancer subtypes on survival of stage III IBC, demonstrated that there is an important association between HR/HER2-based subtypes and response to NACT and OS. Patients with triple-negative IBC have a worse survival, compared to the other subtypes. These results are consistent with previous studies [20], showing that triple-negative tumors have the worst prognosis despite optimal treatment. A recent population-based study demonstrated that triple-negative and HR+/HER2− subtypes are predictors for worse OS in IBC [9]. We confirmed this with respect to triple-negative tumors, but not for HR+/HER2− tumors. This might be due to the fact that in our population endocrine therapy was far more often administered in HR+ patients, in contrast to a US-based population [9].

IBC patients who responded with a pCR after NACT experienced an improved survival compared to non-pCR patients. This improved survival was observed for all subtypes, but especially for HER2+ tumor subtypes. In the present study, the pCR rates in all IBC patients were considerably higher (23.2%) than previously reported in population-based analyses [9, 21]. The HR+/HER2+ and HR−/HER2+ subtypes displayed the highest pCR rates. Likely this reflects the use of HER2-targeted therapies, which was more often applied in our study than in previous studies in case of HER2-positive disease [9, 21].

Our results were comparable with breast cancer in general in which HR−/HER2+ achieved pCR significantly more often than the other subtypes and in which the prognostic impact of pCR was shown to be highest in HER2-positive (non-luminal) and triple-negative tumors [22].

Furthermore, similar pCR rates were found in IBC and NI-LABC, but IBC displayed a lower 5-year OS. These findings suggest that the poor prognosis in IBC might be the result of a higher metastatic risk and other biological features negatively influencing OS [23].

Although previous studies have confirmed the presence of the same subtypes in IBC as originally described in non-IBC, differences do exist: approximately one-third of IBC is HER2+, which is notably higher than non-T4 breast cancer and NI-LABC [3]. Overexpression of HER2 in breast cancer in general is associated with higher recurrence rates and higher mortality, at least in the pre-trastuzumab era [7].

Despite several similarities, the present study has several strengths not found in previous studies concerning IBC. First of all, this study was based on a nationwide population-based cancer registry including unselected and unbiased data of all hospitals (both academic and non-academic) in the Netherlands, compared to mostly single-center studies [14, 24]. Another strength is the inclusion of a very large number of patients, with twice as many patients compared to the largest previously published study [9]. It contains data of very recently treated patients with the incorporation of breast cancer subtypes. This has not been described in many of the previous studies. Furthermore, it also contains data of patients treated with current systemic treatments (including trastuzumab and endocrine treatment) and we have analyzed the influence of trimodality therapy on survival. These issues make the results of our study highly relevant for current daily clinical practice.

However, several limitations of our study have to be discussed. Firstly, the NCR does not register cause of death, and therefore breast cancer-specific survival could not be determined. Secondly, not all patients received surgery, NACT, adjuvant radiotherapy, and/or endocrine treatment in case of HR positivity. While some patients may have refused surgery or treatment was attenuated due to age/comorbidity, it also raises unanswered questions. Decisions concerning treatment strategies and, more importantly, reasons for the waiver of (neo) adjuvant modalities could not be investigated in this database. Treatment recommendations are influenced by the experience of the treating physician and judgment about perceived benefit of the treatment related to the patients’ general condition. Unfortunately, comorbidity was not registered in the NCR. Therefore, we were not able to eliminate it as a potential confounder. We could only account for age: patients receiving NACT were evidently younger compared to patients who did not receive it. This was also seen in the use of trastuzumab: the median age for patients receiving trastuzumab was (54.6 years ± 13.7 years), compared with (63.7 years ± 16.1 years) for patients not receiving it.

Unfortunately, data on risk factors associated with this disease are limited. The NCR does not register ethnicity, BMI, and maternal age at first birth [25, 26].

Furthermore, Ki-67 and tumor grade might be used in the classification of breast cancer subtypes [6, 27]. Compared to non-inflammatory locally advanced breast cancer (NI-LABC), IBC has higher levels of Ki-67, which is associated with worse prognosis [28]. Ki-67 is not registered in the NCR, as it is not routinely assessed in the Netherlands. Moreover, tumor grade was unknown in 65% of patients who underwent NACT. Therefore, assessment of actual tumor grade in relation to treatment effect is difficult. Furthermore, the absence of grade might influence the classification in the afore mentioned tumor subtypes, since high-grade HR+/HER2− tumors can be regarded as luminal B subtypes (59 patients with high-grade HER+/HER2− tumors were classified in the luminal A group but could also be included in the luminal B subtype, Table 1) [29]. However, grade was not included in other, but similar, population-based analyses, rendering results comparable [9].

Unfortunately, information on recurrences was not available for analysis.

Our results suggest that stratification based on breast cancer subtypes in IBC is of clinical use for estimating the rate of pathological response to NACT and OS. This might aid in clinical decision making and counseling patients about anticipated outcomes. However, patients with breast cancer may experience changes in HR/HER2 status and tumor phenotype after NACT. Loss of HR positivity and the switch to the triple-negative phenotype after NACT also are associated with a worse patient outcome [30]. This could not be analyzed because these data were unavailable in the database. Furthermore, a central pathology review during treatment was not conducted, which might have led to an altered receptor status based on IHC in several patients with subsequent changes in systemic treatment [31]. Therefore, the potential impact of inter-institutional discordance was not evaluated. However, our current analysis reflects daily practice in which local laboratories do not send all samples to a central laboratory.

The current management of stage III IBC consists of trimodality therapy and underutilization of trimodality therapy negatively impacts survival in IBC [4, 32]. We also confirmed the positive effect of trimodality therapy on survival. Remarkably, in only 78.0% of patients who were surgically treated trimodality therapy was applied.

Although survival in IBC patients has improved during the last decades, the still dismal prognosis mandates further improvement [3]. It is essential that eligible patients with non-metastatic IBC receive trimodality therapy, since this currently is the most effective regimen. IBC is a heterogeneous disease in which clinical decision making is based on predicting harms and benefits based on both patient and tumor characteristics. Recently, the concept of personalized medicine has taken a more central position in cancer care and advances in technology (e.g., gene sequencing) will allow us to tailor therapies based on this knowledge. Future trials to evaluate molecularly targeted biological agents are necessary to improve survival for patients with this aggressive form of breast cancer. Treatment of IBC will be increasingly based on molecular profiling of tumors, as is the case in all breast cancers, rather than on histology alone. Prognostic and predictive biomarkers are eagerly awaited, especially for triple-negative tumors.

## Conclusion

We showed that, in stage III IBC, HR/HER2-based breast cancer subtypes are important prognostic factors of OS and response to NACT. Patients with triple-negative IBC have the worst prognosis, despite administration of trimodality treatment and reached pCR.

## Data Availability

The data that support the findings of this study are available from the NCR but restrictions apply to the availability of these data, which were used under license for the current study, and so are not publicly available. Data are, however, available from the authors upon reasonable request and with permission of the NCR.
